# 

**Published:** 1902-08-15

**Authors:** 


### CONTENTS.

The Possibilities of Porcelain as a Filling Material,
*By W. T. Reeves, D.D.S.* 379

Suppurative Disease of the Maxillary Sinus,
*By Herbert M. King, M.D.* 403

The X-Rays in Dentistry, - *By P. M. Hickey, M.D.* 421

Editorial, - - - - - - - - 429

Indents, - - - - - - - - - - 430

